# Supplementary material for: Margination and adhesion dynamics of tumor cells in a real microvascular network
Source: PLoS Comput Biol. 2021 Feb 19;17(2):e1008746. doi: 10.1371/journal.pcbi.1008746 (PMC7928530; doi:10.1371/journal.pcbi.1008746)
Supplement: S1 Appendix — (PDF) [file pcbi.1008746.s002.pdf]

Supporting Information: Margination and adhesion dynamics of tumor cells in a real microvascular network

Sitong Wang[1], Ting Ye[1*], Guansheng Li[1], Xuejiao Zhang[1], and Huixin Shi[1]

1 Department of Computational Mathematics, School of Mathematics, Jilin University, Changchun, 130012, China

* yeting@jlu.edu.cn

SDPD-IBM model

The SDPD-IBM formulation is given by,

$$d\boldsymbol{x}_i = \boldsymbol{v}_i dt, \tag{1}$$

$$m d\boldsymbol{v}_i = \boldsymbol{F}_C dt + \boldsymbol{F}_D dt + \boldsymbol{F}_R + \boldsymbol{F}_G dt + \boldsymbol{F}_B dt, \tag{2}$$

$$\boldsymbol{F}_B = \sum_k \beta_{ik}(\boldsymbol{F}_k^{def} + \boldsymbol{F}_k^{agg} + \boldsymbol{F}_k^{adh}), \tag{3}$$

$$\frac{d\boldsymbol{X}_k}{dt} = \sum_i \beta_{ik}\boldsymbol{v}_i. \tag{4}$$

Here, the forces are expressed as follows [1, 2]:

$$\boldsymbol{F}_C = \sum_j \alpha_{ij}\boldsymbol{x}_{ij}, \quad \boldsymbol{F}_D = -\sum_j \gamma_{ij}\left[\boldsymbol{v}_{ij} + (\boldsymbol{e}_{ij}\cdot\boldsymbol{v}_{ij})\boldsymbol{e}_{ij}\right],$$
$$\boldsymbol{F}_R = \sum_j A_{ij}\widehat{d\boldsymbol{W}}_{ij}\cdot\boldsymbol{e}_{ij}, \quad \boldsymbol{F}_G = m\boldsymbol{g}, \tag{5}$$

where $\boldsymbol{v}_{ij} = \boldsymbol{v}_i - \boldsymbol{v}_j$, $\boldsymbol{e}_{ij} = \boldsymbol{x}_{ij}/x_{ij}$, $x_{ij} = |\boldsymbol{x}_{ij}|$ and $\boldsymbol{x}_{ij} = \boldsymbol{x}_i - \boldsymbol{x}_j$. $d\boldsymbol{W}_{ij}$ is a 3×3 matrix for 3D case, constructed by independent increments of the Wiener process, and $\widehat{d\boldsymbol{W}}_{ij}$ is its symmetric part defined as

$$\widehat{d\boldsymbol{W}}_{ij} = \frac{1}{2}\left(d\boldsymbol{W}_{ij} + d\boldsymbol{W}_{ij}^T\right), \tag{6}$$

where $d\boldsymbol{W}_{ij}^T$ is the transposition of $d\boldsymbol{W}_{ij}$. The coefficients $\alpha_{ij}, \gamma_{ij}, A_{ij}$ and β_{ik} are given by

$$\alpha_{ij} = \left(\frac{P_i}{d_i^2} + \frac{P_j}{d_j^2}\right)F_{ij}, \quad \gamma_{ij} = \frac{5\eta}{3}\frac{F_{ij}}{d_i d_j},$$
$$A_{ij} = \left[\frac{20\eta k_B T}{3}\frac{F_{ij}}{d_i d_j}\right]^{\frac{1}{2}}, \quad \beta_{ik} = \frac{W_{ik}}{d_i}, \tag{7}$$

where P_i and $d_i = \rho_i/m$ are the pressure and number density of the fluid particle i, k_BT is the Boltzmann temperature (actually, the specific kinetic energy, to be kept constant), and F_{ij} is the geometrical function defined as

$$F_{ij} = -\frac{1}{x_{ij}}\frac{dW_{ij}}{dx_{ij}}, \tag{8}$$

where W_{ij} is the kernel function, and here we used the cubic spline kernel function given by,

$$W_{ij} = \frac{3}{2\pi h^3}\begin{cases} \frac{2}{3} - \lambda^2 + \frac{1}{2}\lambda^3, & 0 \le \lambda < 1, \\ \frac{1}{6}(2-\lambda)^3, & 1 \le \lambda < 2, \\ 0, & \lambda \ge 2, \end{cases} \tag{9}$$

with $\lambda = x_{ij}/h$ and h is the smoothing length.

Cell deformation model

The deformation energy U^{def} is given by [3],

$$U^{def} = U_s + U_b + U_a + U_v. \tag{10}$$

It is assumed that the triangular network are connect by the worm-like springs, i.e., the triangular edge is modeled as the worm-like spring, and hence the in-plane energy U_s is given by

$$U_s = \sum_{j=1,\cdots,N_s}\left(\frac{k_BTL_j}{4p_j}\frac{3s_j^2 - 2s_j^3}{1-s_j} + \frac{\chi_j}{s_jL_j}\right), \tag{11}$$

where $s_j = l_j/L_j$, l_j and L_j are the current and maximum length of the spring j, k_BT and p_j are the Boltzmann temperature and persistence length, χ_j is a repulsive coefficient, and N_s is the number of springs. The bending energy is defined as

$$U_b = \sum_{j=1,\cdots,N_s} K_B(1 - \cos(\theta_j - \theta_j^d)), \tag{12}$$

where K_B is the bending coefficient, and θ_j and θ_j^d are the current and desired angles between two adjacent triangles having the common edge j. The area-restraint energy consists of the restraint energies from whole cell area and each triangle area,

$$U_a = \frac{K_{AG}(A - A^d)^2}{2A^d} + \sum_{j=1,\cdots,N_t}\frac{K_{AL}(A_j - A_j^d)^2}{2A_j^d}, \tag{13}$$

where K_{AG} and K_{AL} are the global and local area restraint constants, A and A_j are the areas of the whole cell and triangle j, A^d and A_j^d are the corresponding desired areas, and N_t is the number of triangles on the cell membrane. Similarly, the volume-restraint energy is defined as

$$U_v = \frac{K_V(V - V^d)^2}{2V^d}, \tag{14}$$

where K_V is the volume restraint constant, and V and V^d are the current and desired volumes of cell.

Nondimensionalization

To perform the nondimensionalization of theoretical models, three basic characteristic quantities are chosen, that is, using the cut-off radius of SDPD as the basic characteristic length l', the mass of a fluid particle as the basic characteristic mass m', and the Boltzmann temperature of system as the basic characteristic energy ε'. In the present work, we set $l' = 2\mu\text{m}$, $m' = 1.0 \times 10^{-15}\text{kg}$ and $\varepsilon' = 4.142 \times 10^{-21}\text{J}$. Other characteristic quantities can be derived from these three basic ones, *e.g.*, the characteristic time $t' = l'\sqrt{m'/\varepsilon'}$. The physical variables are now scaled with respect to the characteristic quantities, for example,

$$\bar{\boldsymbol{x}}_i = \frac{\boldsymbol{x}_i}{l'}, \quad \bar{\boldsymbol{v}}_i = \frac{\boldsymbol{v}_i}{\sqrt{\varepsilon'/m'}}, \quad \bar{t} = \frac{t}{l'\sqrt{m'/\varepsilon'}} \tag{15}$$

and Eq 1 can be scaled into

$$d\left(\bar{\boldsymbol{x}}_i l'\right) = \left(\bar{\boldsymbol{v}}_i \sqrt{\varepsilon'/m'}\right) d\left(\bar{t} l' \sqrt{m'/\varepsilon'}\right), \tag{16}$$

cancelling out the same quantities from both sides gives

$$d\bar{\boldsymbol{x}}_i = \bar{\boldsymbol{v}}_i d\bar{t}. \tag{17}$$

It is found that the nondimensional form of the SDPD-IBM formulation is the same as its dimensional form. There are several important dimensionless groups associated to the SDPD-IBM model, listed as below, where the subscript '0' refers to the typical values of physical quantities in a given problem.

- Mach number Ma: describes the relative importance of flow inertia to sound speed,

$$\text{Ma} = \frac{v_0}{c}. \tag{18}$$

  Because the SDPD-IBM uses the artificial sound speed to control the fluid compressibility, we always let $Ma \leq 0.1$ in the present work.

- Reynolds number Re: describes the relative importance of flow inertia to viscosity,

$$\text{Re} = \frac{\rho_0 v_0 l_0}{\eta_0}. \tag{19}$$

  In the present work, we let $Re \leq 0.5$, and it has been found that the Reynolds number has a slight effect on the cell behaviors when $Re \leq 1$.

- Froude number Fr: describes the relative importance of flow inertia to gravity,

$$\text{Fr} = \frac{v_0}{\sqrt{g_0 l_0}}. \tag{20}$$

  This number depends on the externally-applied force used to drive the fluid flow.

- Capillary number Ca: describes the relative importance of viscous force to the cell deformation force,

$$\text{Ca} = \frac{\eta_0 v_0}{E_S}, \tag{21}$$

  where E_S is the shear modulus of a cell.

Numerical methods

The velocity-Verlet algorithm is used to solve the SDPD model in Eqs 1-4, and its procedures are given as follows [4, 5].

Step 1: update coordinates of membrane particles

$$\boldsymbol{X}_k^{n+1} = \boldsymbol{X}_k^n + \Delta t \sum_i \beta_{ik}^n \boldsymbol{v}_i^n, \tag{22}$$

where the superscript n refers to the nth simulation step, and Δt is the time step;

Step 2: update coordinates of fluid particles

$$\boldsymbol{x}_i^{n+1} = \boldsymbol{x}_i^n + \Delta t \boldsymbol{v}_i^n + \frac{\Delta t^2}{2m} \boldsymbol{F}_T^n, \tag{23}$$

where $\boldsymbol{F}_T^n$ is the total force;

Step 3: predict velocities of fluid particles

$$\tilde{\boldsymbol{v}}_i^{n+1} = \boldsymbol{v}_i^n + \omega \frac{\Delta t}{m} \boldsymbol{F}_T^n, \tag{24}$$

where ω is an empirical factor with the optimal value of 0.65 [4];

Step 4: calculate all types of forces

$$\boldsymbol{F}_T^{n+1} = \boldsymbol{F}_C + \boldsymbol{F}_D + \boldsymbol{F}_R/\Delta t + \boldsymbol{F}_G + \boldsymbol{F}_B; \tag{25}$$

Step 5: correct the velocities of fluid particles

$$\boldsymbol{v}_i^{n+1} = \tilde{\boldsymbol{v}}_i^{n+1} + \frac{\Delta t}{2m} \boldsymbol{F}_T^{n+1}. \tag{26}$$

After these five steps, the coordinates and velocities of the fluid particles are obtained, as well as the coordinates of the membrane particles of the RBCs and CTCs.

References

1. Español P, Revenga M. Smoothed dissipative particle dynamics. Phys Rev E. 2003;67:1–12. doi:10.1103/PhysRevE.67.026705.

2. Ye T, Phan-Thien N, Lim CT, Peng LN, Shi HX. Hybrid smoothed dissipative particle dynamics and immersed boundary method for simulation of red blood cells in flows. Phys Rev E. 2017;95:063314. doi:10.1103/PhysRevE.95.063314.

3. Fedosov DA, Caswell B, Karniadakis GE. A multiscale red blood cell model with accurate mechanics, rheology, and dynamics. Biophys J. 2010;98:2215–2225. doi:10.1016/j.bpj.2010.02.002.

4. Groot RD, Warren PB. Dissipative particle dynamics: Bridging the gap between atomistic and mesoscopic simulation. J Chem Phys. 1997;107(11):4423–4435. doi:10.3390/ijms14047932.

5. Ye T, Shi HX, Phan-Thien N, Lim CT. The key events of thrombus formation: platelet adhesion and aggregation. Biomech Model Mechanobiol. 2020;19:943–955. doi:10.1007/s10237-019-01262-x.
