# Supplementary material for: Margination and adhesion dynamics of tumor cells in a real microvascular network
Source: PLoS Comput Biol. 2021 Feb 19;17(2):e1008746. doi: 10.1371/journal.pcbi.1008746 (PMC7928530; doi:10.1371/journal.pcbi.1008746)
Supplement: Attachment — Submitted filename: Response to reviewers.pdf [file pcbi.1008746.s003.pdf]

Manuscript Revision Details

Reviewer #1

This is an ambitious multiscale modeling effort of CTC margination and adhesion dynamics that may contribute to the cancer pathophysiology and its therapy. It shows that the RBC hematocrit and blood flow shear rate play key roles in the CTC margination and adhesion behaviors. It is a well-written manuscript that sheds important light on overlooked vascular flow mechanisms that contribute to the tumor metastasis in early stage, some concerns regarding several technical aspects that may affect the simulation results, their validation, and their limitations need to be addressed and/or clarified by the authors.

Comment 1

1) *Adhesion of circulating tumor cells (CTCs) to the microvessel wall largely depends on the blood hydrodynamic conditions, one of which is the blood viscosity. I am wondering whether the authors consider the blood viscosity in their simulations. If yes, can the blood viscosity parameters at different hematocrit compare to experimental values?*

Reply: Thank you for your comments which largely improve the quality of our manuscript. The blood viscosity is considered in our simulations. There are two ways to consider the blood viscosity in the simulations on blood (Ye et al. Phys. Fluids, 31, 011301, 2019). One is to treat the blood as a whole non-Newtonian fluid, and the other is to treat the blood as the group of blood plasma and blood cells. In our work, the second way is used, in which the plasma is a Newtonian fluid, and the blood cells are separately modeled as elastic cells so that the blood viscosity can changes with hematocrit. The comparison of blood viscosity between our numerical results and experimental values has been made in our previously-published work (Ye et al. Phys Fluids., 31:021903, 2019).

In response to this comment, we have added more descriptions about the blood viscosity in the revised manuscript.

- "The whole blood consisting of the plasma and the cells behaves as non-Newtonian fluid, because the cells are incorporated by the singular force ***f***, and thus the blood viscosity is described to depend on the cell hematocrit [58]."

Comment 2

2) *In this study, the simulation results suggest that a moderate hematocrit promotes CTC adhesion, while a relatively low or relatively high hematocrit can help to prevent the adhesion. However, in a previous simulation study (Xiao et al., Biomech Model Mechanobiol, 2017, 16:597-610), it shows that the CTC has an increasing probability of adhesion with the hematocrit due to the increased wall-directed force in small microvessel. Can the authors give some comment on this discrepancy?*

Reply: The hematocrit range in the work of Xiao *et al.* is set as 10-30%. In this range, our results are consistent with the results of Xiao *et al.*, that is, the CTC has an increasing probability of adhesion with the hematocrit due to the increased wall-directed force. However, as the hematocrit is larger than about 30%, the CTC adhesion cannot be promoted any more with increasing the hematocrit, because the wall-directed aggregation force is offset by the

outer RBCs surrounding the CTC. This conclusion was also observed for the white blood cells in previous work (Fedosov et al., Phys Rev Lett. 2012;108:1–5).

In response to this comment, we have added the above discussion in the revised manuscript.

- "From a low hematocrit, such as H_{ct} = 10 - 25%, the radial aggregation force indeed increases as H_{ct} increases to 30%, as shown in Fig. 8(c), which in turn promotes the CTC adhesion. This was also observed in the work of Xiao *et al.* [27], where H_{ct} ranging from 10 to 30% was considered. However, when the hematocrit varies from a normal value to a higher value, for example from H_{ct} = 30 to 40%, the radial aggregation force does not increase but instead decreases, and correspondingly the CTC adhesion becomes weak. This is because the RBC-free layer is narrowed down subject to the more RBCs at a high hematocrit. Thus, the CTC is more likely to be surrounded by those RBCs, as shown in Fig. 8(a), and the outer RBCs give the CTC a center-directed aggregation force, counteracting the wall-directed aggregation force acting on the CTC. This was observed for the leukocytes in the work of Fedosov *et al.* [12]"

Comment 3

3) *In the model setup, the authors state there are 29704 ligand sites on the wall, 1176 receptor sites on the CTC's membrane. Why do they choose these values? Can it compare to experimental data? Also, in general, the experimental measured diameters of CTCs are about 10 ~ 20 microns, which is larger than the CTC diameter used in this study. Why do they choose the parameter of CTC diameter in this way?*

Reply: Thank you very much for your comment. Indeed, these numbers of receptors and ligands are not same as the experimental values. It is extremely challenging for the computer resources to keep the same numbers of receptors and ligands with the experiments and at the same time observe the behaviors of a large number of cells in a microvessels. Hence, most of previous numerical studies adopted the coarse-grained technique. That is, a numerical receptor (ligand) may represent several, tens or hundreds of experimental receptors (ligands). In our work, we also use this technique, and choose the parameter values consistent with the previous simulation studies. For example, the density of ligand sites is 3/μm^2, similar with the work of Zhang et al. (Biomech Model Mechan. 2018;17:223–234.) The number of receptors is 1176, similar with the work of Xiao et al. (Xiao et al. Biomech Model Mechanobiol. 2017;16:597–610.)

In practice, the CTC diameter ranges widely. For example, Gassmann et al. observed that the diameter of a tumor cell in vivo is only about 5μm (Int J Colorectal Dis. 2009;24:851–858), while Guo et al. gave its diameter about 16μm (Biomech Model Mechan. 2014;13:537–550). In simulations, a moderate value about 8-10μm is often chosen for saving computational cost (Yan et al. Biomech Model Mechanobiol. 2012;11:641–653; Xiao et al. Biomech Model Mechanobiol. 2017;16:597–610; Dabagh et al. Cell Mol Bioeng. 2020;13:141–154), and we here choose 9μm in this work.

In response to this comment, we have added the related descriptions and references in the revised manuscript.

- "The RBC is modeled to be a biconcave with a maximum diameter of 7.82 μm [50], while the CTC is spherical with the diameter of 9 μm. Due to the diversity of CTCs, their sizes are very different. In experiments, Gassmann et al. [51] observed that the diameter of a tumor cell in vivo is only about 5 μm, while Guo et al. [34] gave its

diameter about 16 μm. In simulations, a moderate value of 8 - 10 μm is often chosen for saving computational cost [26,27,52].”

- “In the present work, there are 29704 ligand sites on the wall, 1176 receptor sites on the CTC's membrane, and no receptor sites on the RBC's membrane. It should be noted that these numbers are not same as the experimental values due to the limitation of computational resources. Hence, a coarse-grained technique is used, that is, a numerical receptor or ligand may represent several tens or hundreds of experimental receptors or ligands. The number of 29704 ligands is calculated by the fixed number density of 3 μm^{-2}, similar with that used in the work of Zhang *et al.*[55], while the number of 1176 receptors is similar with that used the work of Xiao et al. [27].”

Comment 4

4) *In the Section of "Margination of a CTC in a straight tube", the authors mentioned that the average shear rate is 272 s^{-1}, while in the Section of "Adhesion of a CTC on a plate", the shear rate is about 7000 s^{-1}. Why do they choose two quite different values in the simulations? Also, in the section of "Adhesion of a CTC on a plate", they only simulated the adhesion behavior of a deformable capsule. Since they did not study the adhesion of CTC, I suggest to change the title of this section.*

Reply: The shear rate of 272 s^{-1} is used to keep consistent with the real arteriolar blood flow in microcirculation. The shear rate of 7000 s^{-1} is used to validate the adhesion model, which is same as that used in the work of Zhang *et al.* (Biomech Model Mechan. 2018;17:223–234). In fact, Zhang et al. studied the adhesion of a spherical capsule, instead of a CTC.

In response to this comment, we have changed the section title and also modified the descriptions in the revised manuscript.

- “Adhesion of a deformable capsule on a plate”
- “To fully understand the adhesion behavior and establish reliability of adhesion model, we investigate the adhesion behaviors of a deformable capsule, and compare the simulation results with the work of Zhang et al. [55]”

Comment 5

5) *There are some available work have focused on the modeling of CTC adhesion. Two of them are directly relevant the problem at hand. The authors should reference and comment them to show the novelty of their work.*
1. Xiao et al. (2017), Effects of flowing RBCs on adhesion of a circulating tumor cell in microvessels, Biomech Model Mechanobiol, 16:597-610.
2. Dabagh et al. (2020), Localization of rolling and firm-adhesive interactions between circulating tumor cells and the microvasculature wall. Cell Mol Bioeng, 13:141-154.

Reply: Thank you for your comments. In response to this comment, we have added more literature reviews in the Introduction section, and also highlighted the contributions of the work.

- “However, some experiments [23-25] have shown that the soft CTCs are more likely to extravasate from the circulation, because the soft CTCs have a large contact surface with the vascular wall and thus have a higher possibility of adhesion than the rigid CTCs. Recently, some simulation work [26,27] has also favored the latter conclusion.”

- “Apart from these, some studies [34-36] have shown that the cell adhesion has a considerable dependence on the microvascular geometry. For example, Liu et al. [37] observed in vivo experiments that breast cancer cells preferentially extravasate from the vessel bifurcation. More recently, Hynes et al. [36] and Pepona et al. [38] studied the metastatic behavior within a complex vasculature via both experiments and simulations, and also confirmed that CTCs are prone to attach at the branch points. However, so far there are few studies on the tumor metastasis in a complex microvascular network at a cellular scale, at which the cell deformation, aggregation and adhesion should be taken into account simultaneously [39-41].”

Reviewer #2

In the paper, the authors performed three-dimensional (3D) simulations on the behaviors of the tumor cells in microvascular network and predict the possible CTC adhesion locations. This manuscript exhibits some weaknesses that should be addressed before it can be considered for publication.

Comment 1

1) *The size of the CTC is selected to be spherical with the diameter of 9 micrometer, authors need to provide reference for this size selection, what is the typical size range of CTC in circulation and how does the cell size affect margination, adhesion and the eventual the metastasis.*

Reply: Thank you very much for your comments, which largely improve our quality of the manuscript. In practice, the CTC diameter ranges widely. For example, Gassmann et al. observed that the diameter of a tumor cell in vivo is only about 5μm (Int J Colorectal Dis. 2009;24:851–858), while Guo et al. gave its diameter about 16μm (Biomech Model Mechan. 2014;13:537–550). In simulations, a moderate value about 8-10μm is often chosen for saving computational cost (Yan et al. Biomech Model Mechanobiol. 2012;11:641–653; Xiao et al. Biomech Model Mechanobiol. 2017;16:597–610; Dabagh et al. Cell Mol Bioeng. 2020;13:141–154), and we here choose 9μm in this work.

To our knowledge, there have been few studies about the effect of the CTC size on the margination, adhesion and metastasis. Most of previous studies focused on the deformability of CTC and local hemodynamic conditions. We believe that the CTC size may have a significant effect on its behaviors. For example, a small CTC will stay for a short while at the bifurcation, such that it may be not adhered at that position. We may study this effect in near future. Thanks.

In response to this comment, we have added the above descriptions in the revised manuscript.

- "The RBC is modeled to be a biconcave with a maximum diameter of 7.82 μm [50], while the CTC is spherical with the diameter of 9 μm. Due to the diversity of CTCs, their sizes are very different. In experiments, Gassmann et al. [51] observed that the diameter of a tumor cell in vivo is only about 5 μm, while Guo et al. [34] gave its diameter about 16 μm. In simulations, a moderate value of 8 - 10 μm is often chosen for saving computational cost [26,27,52]."

Comment 2

2) *Again 1176 receptor sites are assigned to the CTC's membrane on what basis and any references for this selections*

Reply: To be honest, the numbers of receptors and ligands are not same as the experimental values. It is extremely challenging for the computer resources to keep the same numbers of receptors and ligands with the experiments and at the same time observe the behaviors of a large number of cells in a microvessels. Hence, most of previous numerical studies adopted the coarse-grained technique. That is, a numerical receptor (ligand) may represent several, tens or hundreds of experimental receptors (ligands). In our work, we also use this technique, and choose the parameter values consistent with the previous simulation studies. For example, the density of ligand sites is 3/μm^2, similar with the work of Zhang et al. (Biomech Model Mechan.

2018;17:223–234.) The number of receptors is 1176, similar with the work of Xiao et al. (Xiao et al. Biomech Model Mechanobiol. 2017;16:597–610.)

In response to this comment, we have added the above descriptions in the revised manuscript.

- "In the present work, there are 29704 ligand sites on the wall, 1176 receptor sites on the CTC's membrane, and no receptor sites on the RBC's membrane. It should be noted that these numbers are not same as the experimental values due to the limitation of computational resources. Hence, a coarse-grained technique is used, that is, a numerical receptor or ligand may represent several tens or hundreds of experimental receptors or ligands. The number of 29704 ligands is calculated by the fixed number density of 3 μm^{-2}, similar with that used in the work of Zhang *et al.*[55], while the number of 1176 receptors are similar with that used the work of Xiao et al. [27]."

Comment 3

3) More important, as showed in the Table 1, the bending and shear stiffness of CTC are smaller than those of red cells, which is unreasonable assumption.

Reply: Due to the diversity of CTCs, their mechanical properties are quite different. Some of them are softer than RBCs, while others are more rigid than RBCs. An optical measurement (Lincoln B et al., Cytom Part A, 2004, 59A:203–209) showed that cancer cells are stretched five times more easily than normal cells. In our work, the values of the bending and shear stiffness of CTC are selected based on the work of Guo et al. (Chinese Phys Lett. 2004;21:2543–2546) and Xiao et al. (Biomech Model Mechanobiol. 2016;15:1655–1667).

In response to this comment, we have added the references for each parameter value in Table 1 in the revised manuscript.

Table 1. Physical quantities and their characteristic quantities.

Physical quantities	Physical values	Characteristic quantities	Simulation values
Fluid density (ρ)	1.0×10^3kg/m^3	m'/l'^3	8
Shear viscosity (η)	1.0×10^{-4}pa · s	$\sqrt{m'\epsilon'}/l'^2$	197
Temperature (T)	300K	ϵ'	1
CTC diameter (D_t)	9.0μm [26]	l'	4.5
RBC diameter (D_r)	7.82μm [50]	l'	3.91
Shear modulus of CTC (E_S)	1.0×10^{-6}N/m [71]	ϵ'/l'^2	9.66×10^2
Shear modulus of RBC (E_S)	6.0×10^{-6}N/m [72]	ϵ'/l'^2	5.794×10^3
Bending modulus of CTC (E_B)	1.35×10^{-19}J [73]	ϵ'	33
Bending modulus of RBC (E_B)	2.07×10^{-19}J [72]	ϵ'	50
Dilation modulus of CTC (E_D)	1.16×10^{-4}N/m†	ϵ'/l'^2	1.12×10^5
Dilation modulus of RBC (E_D)	1.26×10^{-4}N/m†	ϵ'/l'^2	1.22×10^5
Equilibrium length (λ)	0.2μm [55]	l'	0.1
Reactive distance (l_r)	1.0μm [55]	l'	0.5
Adhesion strength (E_A)	3.0×10^{-5}N/m [55]	ϵ'/l'^2	2.9×10^4
Formation strength (σ_f)	6.0×10^{-7}N/m [55]	ϵ'/l'^2	5.8×10^2
Dissociation strength (σ_d)	7.54×10^{-7}N/m [55]	ϵ'/l'^2	7.28×10^2
Unstressed formation rate (k_f^0)	1.205×10^6s^{-1} [55]	$\sqrt{\epsilon'/m'}/l'$	1.205×10^3
Unstressed dissociation rate (k_d^0)	3.55×10^5s^{-1} [55]	$\sqrt{\epsilon'/m'}/l'$	3.55×10^2

† The dilation modulus in simulations is, in general, smaller than real values to save computational cost, as long as it can guarantee that the variation of surface area of a RBC or tumor cell is less than 3%.

Comment 4

4) Details about the adhesion model should be moved to Method section and equations should be provided so that the readers can appreciate the meaning of the all parameters. Also, are those adhesion parameters applicable for modeling CTC, why not use an adhesion model designed specifically for CTC such as the one mentioned by the authors in the introduction section "A stochastic adhesive model was developed by Hammer and Apte [26], which has been widely used to theoretically and numerically investigate the adhesion of various cells, certainly including the CTCs."

Reply: This is a misunderstanding due to our unclear descriptions. The adhesion model we used is exactly the stochastic adhesive model developed by Hammer and Apte.

In order to response to this comment, we have moved the cell models, including the deformation, aggregation and adhesion models to Method section.

- "Discrete elastic model for cell deformation

  To characterize the cell deformation, the cell membrane is modeled as a triangular network by connecting all membrane particles. The edges of each triangle are modeled as springs to describe the membrane elasticity, and the angles of any two neighboring triangles are used to describe the membrane bending. ……….."

- "Morse potential model for cell aggregation

  To describe the cell-cell interaction, the Morse potential model proposed by Liu et al. [66] is used, in which the total aggregation energy between the cells is approximated as [67],………………."

- "Stochastic binding model for cell adhesion

  To characterize the CTC adhesion, the stochastic binding model proposed by Hammer and Apte [28] is used, in which an adhesion potential energy is given by,……………."

Comment 5

5) It is not clear the adhesion of the CTC at the bifurcation is permanent or temporary, if the CTC is flowing into a vessel with a size smaller than the CTC, could it block the vessel?

Reply: Thank you very much. That is a great comment. Whether the CTC adhesion is permanent or not depends on the number of receptor-ligand bonds that is formed stochastically. If the adhesion strength is set to be strong enough, there are a large number of bonds formed, the CTC experiences a firm adhesion. In such case, the vessel is easily blocked due to the CTC adhesion. However, in our work, we do not set such strong enough adhesions strength, and hence the CTC is not firmly adhered so that the vessel is not blocked completely. This is observed from the S1 video of Supporting information, as well as confirmed by the video of in vivo observation of Liu et al. (Liu et al., Science, 2018, 360: eaaq1392).

In response to this comment, we have added the above descriptions in the revised manuscript.

- "After the CTC passes through the bifurcation, the bond number quickly decreases to zero while the velocity of CTC becoming faster. It is clear that this CTC does not experience a firm adhesion, and thus its adhesion is temporary."

Comment 6

6) *Authors only test three cases of hematocrit 20% 30% and 40% to draw the conclusion that CTC adhesion is more prominent at moderate hematocrit. This conclusion is not convincing. More cases of hematocrits should be examined considering the variation of hematocrit in the microcirculation is wide.*

Reply: In practice, the hematocrit of human blood is about 30%. The value of 40% is a very high hematocrit. As shown in Fig. 4, even for 40% there are a lot of RBCs filled in the microvessel. Hence, there is no need to check the value beyond 40%, but we have extended the lower bound to 10%, and also added the hematocrit of 15%, 25% and 35%.

In response to this comment, we have added more simulation cases to better support the results in the revised manuscript.

- "Fig. 8 shows the effect of the RBC hematocrit, which is adjusted to be H_{ct}=10 - 40% by varying the number of RBCs in the microvascular inlet. The fluid flow has a mean velocity of $\bar{v}_m$ of 0.735 in all these cases. It is found that the bond number at H_{ct} ≥30% is obviously greater than that at H_{ct} < 30%. Its peak value is larger than 11 for H_{ct} ≥30%, but between 4 to 7 for H_{ct} < 30%, as shown in Fig. 8(b). This implies that the CTC has the strongest adhesion at H_{ct} = 30%, and this is also supported by the radial aggregation force that achieves the maximum value at H_{ct} = 30%, as shown in Fig. 8(c).

Comment 7

7) *The key findings of this work is "These results suggest that the tumor cells may be more likely to extravasate at the microvascular bifurcations if the blood flow is slow and the hematocrit is moderate." Could the authors provide any clinical evidence to back up or correlate with this finding. This should be added to the discussion section which is basically repeating the result section in the current form.*

Reply: There are some experimental phenomena that can support this main conclusion. Liu et al. (Science. 2018;360:eaaq1392) found that MDA-MB-231 human breast cancer cells in vivo adhere at the microvascular bifurcation, and then extravasate from this position. Further, in vivo experiment of Guo et al. (Biomech Model Mechan. 2014;13:537–550) also indicated that the tumor cells may more often adhere at the bifurcated regions of vessels, as well as small venules with relatively low blood flow rates.

In response to this comment, we have added these clinical evidences in the revised manuscript.

- "This again confirms that the CTC is more likely to be adhered at the position of a bifurcation. The similar phenomena have been observed in experiments. Liu et al. [37] found that MDA-MB-231 human breast cancer cells adhere at the microvascular bifurcations, and then extravasate from the blood vessel, by an in vivo experiment. Guo et al. [34] also observed that the tumor cells may more often adhere at the bifurcated regions of vessels, as well as small venules with relatively low shear rates."

Comment 8

8) *Also the authors should could review some literature on inhibiting of tumor metastasis through predicting the location where tumor cells extravasate from the circulation to strengthen the clinical significance of this work.*

Reply: Thank you so much for your comments. In response to this comment, we have added the related references in the revised manuscript.

- "Apart from these, some studies [34-36] have shown that the cell adhesion has a considerable dependence on the microvascular geometry. For example, Liu et al. [37] observed in vivo experiments that breast cancer cells preferentially extravasate from the vessel bifurcation. More recently, Hynes et al. [36] and Pepona et al. [38] studied the metastatic behavior within a complex vasculature via both experiments and simulations, and also confirmed that CTCs are prone to attach at the branch points. However, so far there are few studies on the tumor metastasis in a complex microvascular network at a cellular scale, at which the cell deformation, aggregation and adhesion should be taken into account simultaneously [39-41]."

Reviewer #3

Our understanding of cancer metastasis is directly tied to knowing how and why CTCs will adhere and marginate. The work here is targeting these issues using a 3D computational model. They confirm a higher likelihood of adherence at bifurcations and investigate the influence of nearby RBCs. The authors suggest that CTCs are more likely to extravasate at bifurcations if the flow is slow and hematocrit moderate. They suggest the work provides near insights into cancer pathophysiology and its diagnosis and therapy. Overall the topic of the paper is interesting but it seems to need further data to really be able to make statistically significant conclusions from the experiments proposed.

Comment 1

1) *The authors rightly point out that the origin of such studies comes from leukocyte literature. They mention on line 64 that the model from Hammer and Apte has been used for CTC models but a citation would strengthen this. When discussing prior work, Au et al in PNAS, Takeishi's CTC model, Dabagh et al's adhesion model, and then the work from Hynes and Pepona in CMBE and Science Advances show simulations in complex microvascular networks. Particularly the latter explore the idea of CTC adhesion at bifurcations. Differentiating from these works is important.*

Reply: Thank you very much for your comments, which largely improve our quality of the manuscript. In response to this comment, we have made a more literature review, and added the related references in the revised manuscript.

- "A stochastic adhesive model was developed by Hammer and Apte [28], which has been widely used to theoretically and numerically investigate the adhesion of various cells, certainly including the CTCs [27]."
- "Apart from these, some studies [34-36] have shown that the cell adhesion has a considerable dependence on the microvascular geometry. For example, Liu et al. [37] observed in vivo experiments that breast cancer cells preferentially extravasate from the vessel bifurcation. More recently, Hynes et al. [36] and Pepona et al. [38] studied the metastatic behavior within a complex vasculature via both experiments and simulations, and also confirmed that CTCs are prone to attach at the branch points. However, so far there are few studies on the tumor metastasis in a complex microvascular network at a cellular scale, at which the cell deformation, aggregation and adhesion should be taken into account simultaneously [39-41]."
- "In the present work, we implement the direct three-dimensional modeling of tumor metastasis in a real and complex microvascular network extracted from the mesenteric microvessels of a rat. The deformation, aggregation and adhesion behaviors of cells are considered so as to deeper investigate the margination and adhesion dynamics of tumor cells."

Comment 2

2) *Why was a rat mesentery geometry chosen? Is it representative of a human's? How does the ratio of cell size to vessel diameter compare? If we're to make conclusions about human CTCs based on this, further justification/discussion is needed. Further, for the geometry, how was the geometry obtained and segmented? This work seems to lack details needed to understand the method. What was the adjustment made to achieve one outlet? Why was this needed? Are the CTCs introduced so that they don't interact? The number of CTCs in this simulation as shown in Fig 1B seems unphysiologically high. It looks like*

some of the branches are nearing a diameter of 9 microns (ie similar or smaller to the CTC). If the branch size gets smaller than the CTC, can you really neglect the cell nucleus?

Reply: The present work focuses on the CTC behaviors in a complex microvascular network. For the numerical simulations, the configuration of the microvascular network is not important. Even using a human microvessel, we can only say that the conclusions are true for the selected microvessels. For other human vessels, the conclusions may be not true. Hence, in the present work, we reproduced the microvascular network of a rat mesentery, because it was clearly constructed in the work of Zakrzewicz et al. (News Physiol Sci. 2002;17:197–201)

We use the commercial software ”SOLIDWORKS” to reproduce this microvascular network. The diameters of each microvessels range from 6.4-16μm, and follows Murray’s law, a general law for a large number of transport networks, such as the vascular systems of animals, xylem in plants, and the respiratory system of insects.

One outlet is just for saving the computational cost when imposing the inflow and outflow boundary conditions in simulations. In fact, we have not no need to fix only one outlet, and see our previously-published work (Li et al., J. Comput. Phys, 2020, 401:109031)

Physiologically, there is only one tumor cell per 10 billion red blood cells. Such a large-scale simulation is almost impossible for the nowadays computer. Hence, we put one CTC every 13 RBCs.

The CTC nucleus is not included in the present work, which is a limitation of our cell model. We believe that if the branch size is far less than the CTC, there should be have some effect on the CTC behaviors with and without a nucleus.

In response to this comment, we have added more descriptions about the construction of the microvascular network, the boundary conditions of the models, and the cell models in the revised manuscript.

- “A mesenteric tumor is taken for example in the present work, and it has been found in all age groups from infancy to the very elderly [48]. Fig. 1(a) shows a segment of real microvessels in the rat mesentery [47]. One can consider human microvessels, and there are no large differences for numerical simulations, except that the vessel configurations are required to be reconstructed. In simulations, we choose a portion of this segment to construct microvascular network model by using the commercial software ``SOLIDWORKS". This network is geometrically characterized by branching, merging and tortuous microvessels, and a small adjustment is made for getting only one outlet to suit the inflow and outflow boundary conditions used, as shown in Fig. 1(b). The length of the network is 260 μm, and the microvessels have circular cross section with the diameters ranging from 6.4 to 16 μm. Note that the diameter of each microvessel is calculated by Murray's law [49], a general law for a large number of transport networks, such as the vascular systems of animals, xylem in plants, and the respiratory system of insects.”
- “The CTC’s nucleus is also neglected for the same purpose, saving the computational cost, and this is one of limitations of our cell model.”

Comment 3

3) *I'm confused about the first set of simulations. Typically when investigating cells in flow, you run the simulation through an initialization phase so that the cells are found throughout the geometry. Then you can start studying the behavior of the CTCs. In this case, the authors are studying what happens as the geometry fills. This doesn't seem realistic and could lead to behavior and interactions that are not representative of realistic cases. Why are measurements being made before some sort of convergence in Hct? They mention the CTCs moving away from the centerlines but it is difficult to quantify this here. This figure seems to support what has been shown often for leukocytes and was recently highlighted by Pepona et al, that CTCs will likely adhere at bifurcations. It's good to confirm it but hard to identify the novel contribution in this particular study.*

Reply: This is a misunderstanding due to our unclear descriptions. In the first set of simulations, we use the inflow/outflow boundary condition, which is different the periodic boundary condition that is often used in the previously-published studies. Actually, this is one of advantages of our models (Li *et al.*, J. Comput. Phys, 2020, 401:109031). This boundary condition can ensure that the system including cells and fluid reach a steady state when they flow into the microvascular network.

We have added an index, the distance from the CTC center to the vessel wall, to quantify the margination behavior in the revised manuscript.

In the present work, we mainly perform 3D numerical simulation of cells in a complex microvascular network, and analyze the effects of hematocrit of RBCs and shear rate of blood flow, to understand the CTC margination and adhesion. This work includes the deformation of RBCs and CTCs, interaction among RBCs and CTCs, and the adhesion of CTCs. To our knowledge, there have been few simulation studies done on these behaviors in a complex microvascular network at the same time.

In response to this comment, we have added the quantitative descriptions in the revised manuscript.

- "The inflow/outflow boundary condition is applied in the inlet/outlet of microvascular network, also for two purposes. One is to keep cells continuously moving into the microvascular network, and the other is to settle down the fluid and membrane particles that move out from the microvascular network. This boundary condition is one of advantages of our model, which not only ensures that the system including cells and fluid have reached steady states when they flow into the microvascular network, but also guarantees that the mass and momentum of the system are conserved. More details about this boundary condition can be found in our previous work [59]."
- "It is calculated that the distance between the CTC-5 centroid and the vessel wall ranges from 3.85 to 1.71 as the vessel diameter ranges from 7.7 to 5.57. That is, the CTC is deviated from the centerline of the vessel towards the wall, such that it arrives at the vessel wall completely. The similar margination behavior occurs with other CTCs, which provides more opportunities for the adhesion. The marginated CTC-5, CTC-6, CTC-7 are observed in Fig. 2. Therefore, the margination is often regarded as a prerequisite for the adhesion, especially in the larger vessels."
- "In the present work, we implement the direct three-dimensional modeling of tumor metastasis in a real and complex microvascular network extracted from the mesenteric microvessels of a rat. The deformation, aggregation and adhesion behaviors of cells are considered so as to deeper investigate the margination and adhesion dynamics of tumor cells."

Comment 4

4) What are the effects of the initial RBC placement? It seems like for Figure 4, this simulation is only completed once so it is hard to isolate the influence of the RBC placement. Showing the results under different instantiations would help discern the impact from the radial aggregation force. It's great to be including results with the red blood cells vs just a CTC in the tube, but further information is really needed to sort out the impact of the RBC interactions. One of the conclusions relies on the CTC still being surrounded by RBCs at the end and suggests this is why you don't see adhesion in a straight tube. This conclusion seems like a jump when looking at only one instantiation for a short period. If the simulation were to run longer (or in a longer tube) what would happen with the CTC near the wall? Would it continue to push toward the wall and make more contact or continue to be surrounded by RBCs? If the latter, why? There is also a discussion of shear rate in the margination section without simulation data for multiple shear rates, so it's unclear how any conclusions about the impact of low shear can be drawn. From the methods section, it doesn't look like there is an inter particle potential implemented? Interaction between individual RBCs and the RBC/CTC needs to be taken into account and can influence the CTC trajectory.

Reply: Thank you for your comments. In this simulation case (the second set of simulations), the periodic boundary condition is adopted, the cells have passed the tube for a total of 12 cycles rather than once only. Therefore, the effect of the initial RBC placement has been cancelled out.

We have added one more case with the low shear rate, to better discern the impact from the radial aggregation force, and thus study the difference under the high and low shear rates. In the case of the low shear rate, the margination is observed to be more obvious. For this case, the simulation is also run for a total of 12 cycles, which spent 18 days due to the low flow velocity.

There is definitely the interaction between the cells, which is described by the Morse potential model.

In response to this comment, we have added the more details about the simulation conditions and cell aggregation model, and also added one more simulation case with the low shear rate.

- "The accelerations of $\bar{\boldsymbol{g}} = 19.15$ and 5.73 are applied to generate the fluid flow with the different mean velocities of $\bar{\boldsymbol{v}}_m = 2.94$ and 0.88, respectively. Thus, the average shear rates are calculated to be $\bar{\dot{\gamma}} = \bar{v}_m / \bar{D}_v = 0.27$ and 0.08, corresponding to the physical values of 272 s^{-1} and 81 s^{-1}. They are regarded as a high shear rate for the real arteriolar flow and a low shear rate for the venular flow in microcirculation, respectively [42,56]. In addition, we use a periodic boundary condition in consideration of the simple straight cylindrical tube, such that a long simulation is allowed to be run. In simulations, the cells pass through the tube for 12 rounds, and thus the effect of the initial configurations of the cells can be negligible."
- "Morse potential model for cell aggregation

  To describe the cell-cell interaction, the Morse potential model proposed by Liu et al. [66] is used, in which the total aggregation energy between the cells is approximated as [67],………………."

- “At the initial state, the CTC is placed at the tube centerline, while the RBCs are evenly distributed throughout the tube, as shown in Fig. 4(a). As the time elapses, the RBCs gradually migrate towards to the tube centerline, and the RBC-free layer is presented around the tube wall, as shown in Fig. 4(b). Meanwhile, the CTC is expelled away from the tube centerline, and an obvious margination behavior is observed in Fig. 4(c). This margination behavior is mainly attributed to the aggregation force acting on the CTC from the surrounding RBCs. Fig. 5 shows the deviation of the CTC centroid from the tube centerline, and the radial aggregation force acting on the CTC from the RBCs. It is easily found that they are directly related. When the radial aggregation force is larger than zero, i.e., it is wall-directed, the deviation of the CTC centroid from the tube centerline continuously increases, that is, the CTC is pushed towards the vessel wall. In contrary, when this force is less than zero, i.e., it is center-directed, the CTC moves towards the tube centerline. Taking $\bar{\dot{\gamma}}$ = 0.08 for example, as $\bar{X}_c \in [0, 70]$, the radial aggregation force is larger than zero, and thus the CTC continuously deviates from the tube centerline. As $\bar{X}_c \in [70, 120]$, the radial aggregation force is smaller than zero, and thus the CTC moves towards to the tube centerline.

  Another phenomenon is also observed from Fig. 5 that as the CTC gets closer to the wall, it is pushed back towards the tube centerline to some extent. Hence, the CTC cannot, in fact, approach to the vessel wall enough to exhibit the adhesion behavior, albeit the margination observed. We call this the incomplete margination, for example, the margination of CTC at $\bar{X}_c = 70$ under $\bar{\dot{\gamma}} = 0.08$, and the margination of CTC at $\bar{X}_c = 190$ under $\bar{\dot{\gamma}} = 0.27$. This is attributed to the local interaction between the CTC and the RBCs near the wall. As the CTC is close enough to the vessel wall, it is still surrounded by several RBCs. These RBCs provide a relatively strong center-directed aggregation force, because the distance between the CTC and RBCs is small enough at this moment. This force pushes the CTC back to the tube centerline to some extent.

  In addition, it is also found from Fig. 5 that the wall-directed force at $\bar{\dot{\gamma}} = 0.08$ is less than that at $\bar{\dot{\gamma}} = 0.27$ on the whole, but the CTC margination at $\bar{\dot{\gamma}} = 0.08$ is more obvious. This is because the wall-directed force acts on the CTC much longer at the low shear rate than at the high shear rate. As a result, the CTC margination often occurs in the venules where the blood flows slowly although the wall-directed force is small, rather than the arterioles with a high shear rate. That is one of the reasons why the CTC adhesion is more likely to occur in the venules and rarely in the arterioles [34]. Certainly, if the vessel is narrow enough, such as the capillary in Fig. 2(d), the CTC can reach the microvascular wall completely, and its adhesion may happen.”

Comment 5

5) *The adhesion work recreates the setup of Zhang et al. While the parameters are well described, there motivation seems lacking. Is it just because that is what Zhang et al did? Why is this representative of the question at hand? Much of this section is just recapitulated the prior results from [46]. The biggest difference seems to come from the bond number and it is suggested this is due to the deformation model used. Is a strain hardening or strain softening model more representative of the CTCs at hand? How should we interpret this change? It’s been shown in many previous papers that deformability,*

adhesion, and shear fluid influence adhesion. It's hard to discern in this section what contribution is being added.

Reply: The purpose of this part is to validate the reliability of our adhesion model. For the deformation model, it is quite difficult to determine which model of strain hardening or strain softening is more representative for a CTC, because of the diversity of CTCs.

In response to this comment, we have changed the title of this section in the revised manuscript.

- "Adhesion of a deformable capsule on a plate "
- "To fully understand the adhesion behavior and establish reliability of adhesion model, we investigate the adhesion behaviors of a deformable capsule, and compare the simulation results with the work of Zhang et al. [55]"

Comment 6

6) *For the bifurcation work, they look at three different hematocrit levels. How were 20/30/40 chosen? I wonder if the 10% jump is too much? Will you see those levels in the microvasculature regions being looked at? Is this physiologically relevant? Again, it seems like only one simulation is completed so it's hard to tell how much the initial position and orientation of the red blood cells influence. From (a) of Figure 8, it looks like you have artifacts from the setup phase before the CTC even hits the bifurcation which could influence it's interaction. Typically we would see the simulation run to a steady state in terms of deformation of the nearby cells before measuring the effect on a CTC of interest. It's difficult to make any serious conclusions from the singly simulation without getting past these initialization artifacts. Moreover, what more is added over the recent work from Pepona et al. in CMBE?*

Reply: In practice, the hematocrit of human blood is about 30%. To give more reasonable results, we not only extend the lower bound to 10% from 20%, but also refine the jump interval by 5%.

Same as the reply of Comment 3, the boundary condition is the inflow/outflow boundary condition for this case, and also use a very long inlet to get a high Hct, as shown in the following figure. Therefore, the effects of the initial position and orientation of the RBCs should be negligible.

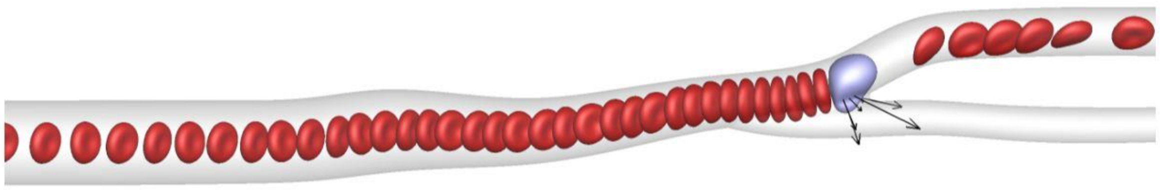

In response to this comment, we also have added more simulation cases with the Hct jump of 5%, and described the work of Pepona et al. in CMBE in the Introduction.

- "
- Fig. 8 shows the effect of the RBC hematocrit, which is adjusted to be $H_{ct} = 10 - 40\%$ by varying the number of RBCs in the microvascular inlet. The fluid flow has a mean velocity of $\bar{v}_m$ of 0.735 in all these cases. It is found that the bond number at $H_{ct} \geq 30\%$

is obviously greater than that at $H_{ct} < 30\%$. Its peak value is larger than 11 for $H_{ct} \geq 30\%$, but between 4 to 7 for $H_{ct} < 30\%$, as shown in Fig. 8(b). This implies that the CTC has the strongest adhesion at $H_{ct} = 30\%$, and this is also supported by the radial aggregation force that achieves the maximum value at $H_{ct} = 30\%$, as shown in Fig. 8(c)."

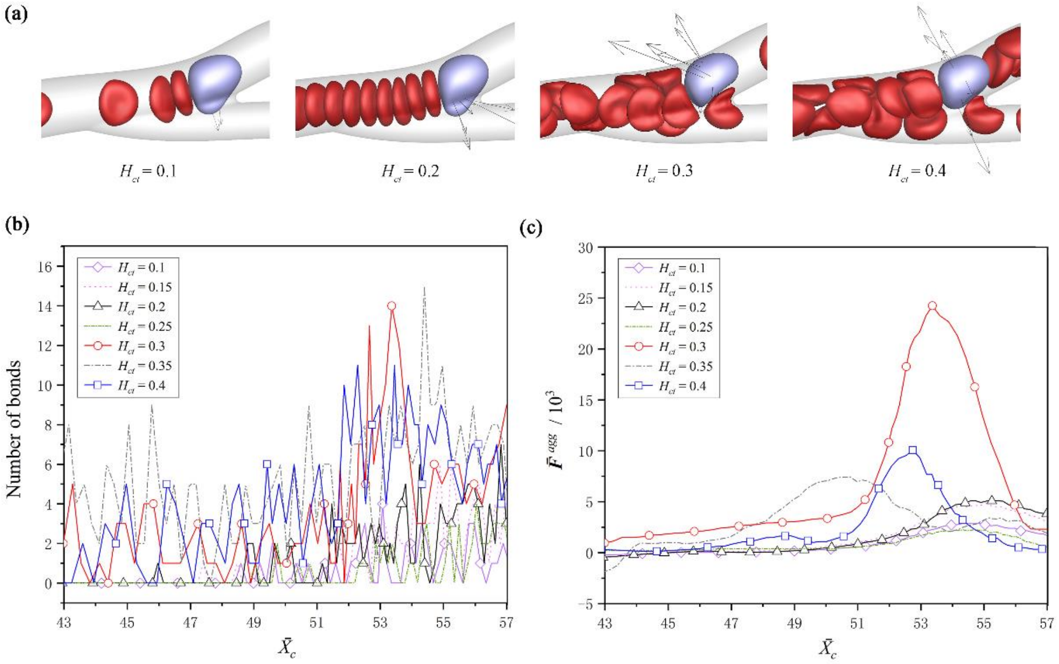

Fig 8 Effect of the RBC hematocrit on the CTC adhesion.

Comment 7

7) *The author summary could be edited for clarity/English in a few places. For example "Thus, what conditions do the tumor cells happen metastasis easily, and where do they extravasate from easily?" needs editing.*

Reply: Thank you for your comments. In response to this comment, we have checked the whole manuscript, and especially modified the unclear descriptions in the author summary.

- "Cancer is one of leading causes of death in the world, but unfortunately, the mechanism of tumor metastasis remains unclear so far. Intuitively, the tumor metastasis starts from a tumor cell migrating towards the vessel wall (namely margination), then adhering to the vessel wall (namely adhesion), and finally extravasating from where it adheres onto. Hence, it is important to investigate the margination and adhesion of tumor cells for understanding the tumor metastasis. We implemented a three-dimensional simulation to directly reproduce these two processes at a cellular scale, where the dynamic behaviors, such as deformation, aggregation and adhesion, of a lot of cells in a very complex microvascular network are taken into account. The results suggest that the tumor cells may be prone to adhere at the microvascular bifurcations with low shear rate and moderate hematocrit, because of a high wall-directed force from the surrounding RBCs. …"
